# Prevalence of asthma symptoms based on the European Community Respiratory Health Survey questionnaire and FENO in university students: gender differences in symptoms and FENO

**DOI:** 10.1186/1710-1492-7-15

**Published:** 2011-09-19

**Authors:** Tamotsu Ishizuka, Shinichi Matsuzaki, Haruka Aoki, Masakiyo Yatomi, Yosuke Kamide, Takeshi Hisada, Takahiro Tsuburai, Kunio Dobashi, Kihachi Ohshima, Kazuo Akiyama, Masatomo Mori

**Affiliations:** 1 Department of Medicine and Molecular Science, Gunma University Graduate School of Medicine, 3-39-15 Showa-machi, Maebashi 371-8511, Japan; 2 Clinical Research Center for Allergy and Rheumatology, National Hospital Organization, Sagamihara National Hospital, 18-1 Sakuradai, Sagamihara, Kanagawa 228-8522, Japan; 3 Gunma University Graduate School of Health Sciences, 3-39-15 Showa-machi, Maebashi 371-8511, Japan; 4 Health and Medical Center, Gunma University, 4-2 Aramaki-machi, Maebashi 371-8510, Japan

## Abstract

**Background:**

The fractional concentration of nitric oxide in exhaled air (FENO) is used as a biomarker of eosinophilic airway inflammation. FENO is increased in patients with asthma. The relationship between subjective asthma symptoms and airway inflammation is an important issue. We expected that the subjective asthma symptoms in women might be different from those in men. Therefore, we investigated the gender differences of asthma symptoms and FENO in a survey of asthma prevalence in university students.

**Methods:**

The information about asthma symptoms was obtained from answers to the European Community Respiratory Health Survey (ECRHS) questionnaire, and FENO was measured by an offline method in 640 students who were informed of this study and consented to participate.

**Results:**

The prevalence of asthma symptoms on the basis of data obtained from 584 students (266 men and 318 women), ranging in age from 18 to 24 years, was analyzed. Wheeze, chest tightness, an attack of shortness of breath, or an attack of cough within the last year was observed in 13.2% of 584 students. When 38.0 ppb was used as the cut-off value of FENO to make the diagnosis of asthma, the sensitivity was 86.8% and the specificity was 74.0%. FENO was ≥ 38.0 ppb in 32.7% of students. FENO was higher in men than in women. The prevalence of asthma symptoms estimated by considering FENO was 7.2%; the prevalence was greater in men (9.4%) than women (5.3%). A FENO ≥ 38.0 ppb was common in students who reported wheeze, but not in students, especially women, who reported cough attacks.

**Conclusions:**

The prevalence of asthma symptoms in university students age 18 to 24 years in Japan was estimated to be 7.2% on the basis of FENO levels as well as subjective symptoms. Gender differences were observed in both FENO levels and asthma symptoms reflecting the presence of eosinophilic airway inflammation.

**Trial registration number:**

UMIN000003244

## Background

Bronchial asthma is a chronic inflammatory disease characterized by reversible airway limitation and airway hyper-reactivity. Clinically, patients with asthma have repeated symptoms, such as wheeze, shortness of breath, or cough, especially at night or early morning. When we make a diagnosis of asthma, it is helpful to expect that the patients have eosinophilic airway inflammation although asthma is not always associated with eosinophilic inflammation. In recent years, the fractional concentration of nitric oxide in exhaled air (the fraction of exhaled nitric oxide, FENO) has been used as a biomarker of eosinophilic inflammation in the airway [1-3]. In fact, FENO is increased in asthmatic patients compared to healthy subjects [4]. Measurements of FENO are useful for making the diagnosis of asthma, and its sensitivity and specificity as a marker for the diagnosis are relatively high [5-8]. There are two procedures for FENO measurements: online and offline. Exhaled gas is collected in a reservoir and subsequently analyzed for nitric oxide (NO) concentrations in offline measurements [9]. Particularly in epidemiologic studies, offline measurement might be superior to online measurement because it is economical and makes it easy to measure a number of samples in a short time [10,11].

Worldwide epidemiological surveys on adult asthma have been mainly performed using the European Community Respiratory Health Survey (ECRHS) questionnaire [12-17]. Asthma symptoms including wheezing, a nocturnal feeling of tightness in the chest, a nocturnal attack of shortness of breath, or a nocturnal attack of cough in the ECRHS questionnaire are consistent with asthma, but certainly not specific for asthma. Moreover, we cannot guess the degree of eosinophilic airway inflammation in each subject based on the information obtained from a questionnaire. Therefore, we think that the combination of FENO measurements and the questionnaire about asthma symptoms might make it possible to develop a more objective estimate in epidemiological studies of asthma prevalence.

In this study, an analysis of the prevalence of asthma symptoms in university students was attempted using both the ECRHS questionnaire and FENO measured by an offline method. Additionally, we tried to investigate the gender differences in FENO levels and asthmatic symptoms.

## Methods

### 1. Subjects

**Figure 1:**
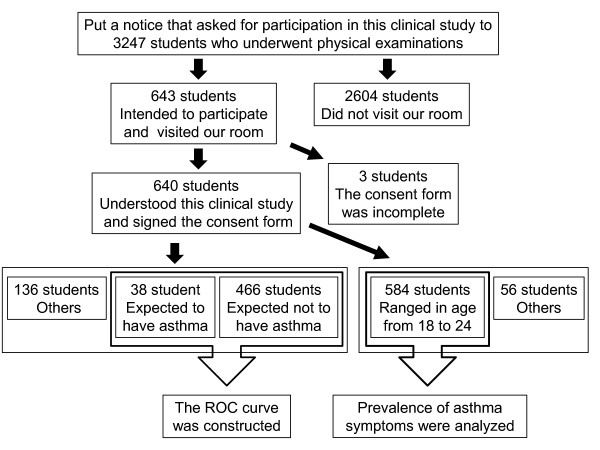
**The flowchart of this clinical study**. We explained this clinical study in detail using brochure for 643 students who intended to take part in our clinical study and visited the room. We got the consent forms adequately signed by 640 students. In order to investigate the prevalence of asthma symptoms, we analyzed the information obtained from the ECRHS questionnaire and FENO data in 584 students whose ages were from 18 to 24.

This study was approved by the Institutional Review Board of Gunma University Hospital, on January 27, 2010 and registered with UMIN-CTR on February 13, 2010 (ID number: UMIN000003244). Gunma University students who participated in the study were asked to fill out an ECRHS questionnaire (Japanese version) [18], and breath samples were obtained. The study was performed from April 1 to 9, 2010 when physical examinations for students were done. The flowchart of this clinical study is shown in Figure 1. We put up a notice that asked for participation in our clinical study. Of the 3247 students who underwent physical examinations, 643 students intended to take part in this study and visited our room. These 643 students were informed of the study in detail. The 640 students consented to participate in the study and signed the consent form. The receiver operating characteristic (ROC) curve was produced using FENO data obtained from 504 samples out of 640 samples (Additional file 1). Samples obtained from 38 students who had suffered from asthma and had wheezing for the last year were used as samples from an asthma group, and samples obtained from 466 students who had not suffered from asthma, did not have any asthmatic symptoms, and had not experienced chronic bronchitis-like symptoms were used as samples from a non-asthma group. When 38.0 ppb was used as the cut-off value to classify the subjects in this study, the sensitivity was 86.8% and the specificity was 74.0%. To investigate the prevalence of asthma symptoms in the specified generation, we used the data obtained from 584 Gunma University students (266 male and 318 female students; age range, 18 to 24 years; mean ± SD age, 19.6 ± 1.6 years) and did not use the data from 56 students who were ≥ 25 years old in this investigation. The prevalence of asthma symptoms was analyzed based on the previous 1-year period.

### 2. Methods

The original 9 main questions containing sub-questions translated into Japanese in the Japanese version of the ECRHS a 1-page questionnaire were used in this study [18,19]. Breath samples were obtained using an offline kit produced by the Center for Environmental Information Science (Tokyo, Japan) to measure FENO under the same conditions as with online measurement [10,11]. In this device, the expiratory flow rate is adjusted to 50 mL/s when subjects exhale at 1.5 kPa oral pressure after inhaling deeply. The adjustment was performed with a variable flux pump (SIBATA, Soka, Japan). A disposable paper mouthpiece and a viral and bacterial filter were connected to a plastic mouthpiece adaptor with a pressure gauge, followed by a plastic T-tube with a resistance valve and a bag reservoir for collecting exhaled gas. A 1.5-L Mylar bag (Sievers, Boulder, CO) was used for the reservoir bag. During expiration, subjects were asked to maintain a constant mouth pressure (1.5 kPa) by looking at a pressure gauge. Air exhaled in the first 5 seconds (250 mL) was discarded, and the air exhaled between 5 to 10 seconds (250 mL) was collected in the Mylar bag. Samples obtained in bags were stored at 4°C to overcome error induced by sample storage and FENO was measured within 8 hours using a chemiluminescence analyzer (NOA 280, Sievers).

### 3. Statistical analysis

Statistical analysis was performed using SPSS software (SPSS Japan Inc., Tokyo, Japan). Differences in percentages between two groups were analyzed using the chi-square test, and differences in means between two groups were analyzed using Student's *t*-test.

## Results

### 1. Gender differences in FENO and symptoms, especially wheeze and nocturnal cough attacks

**Figure 2:**
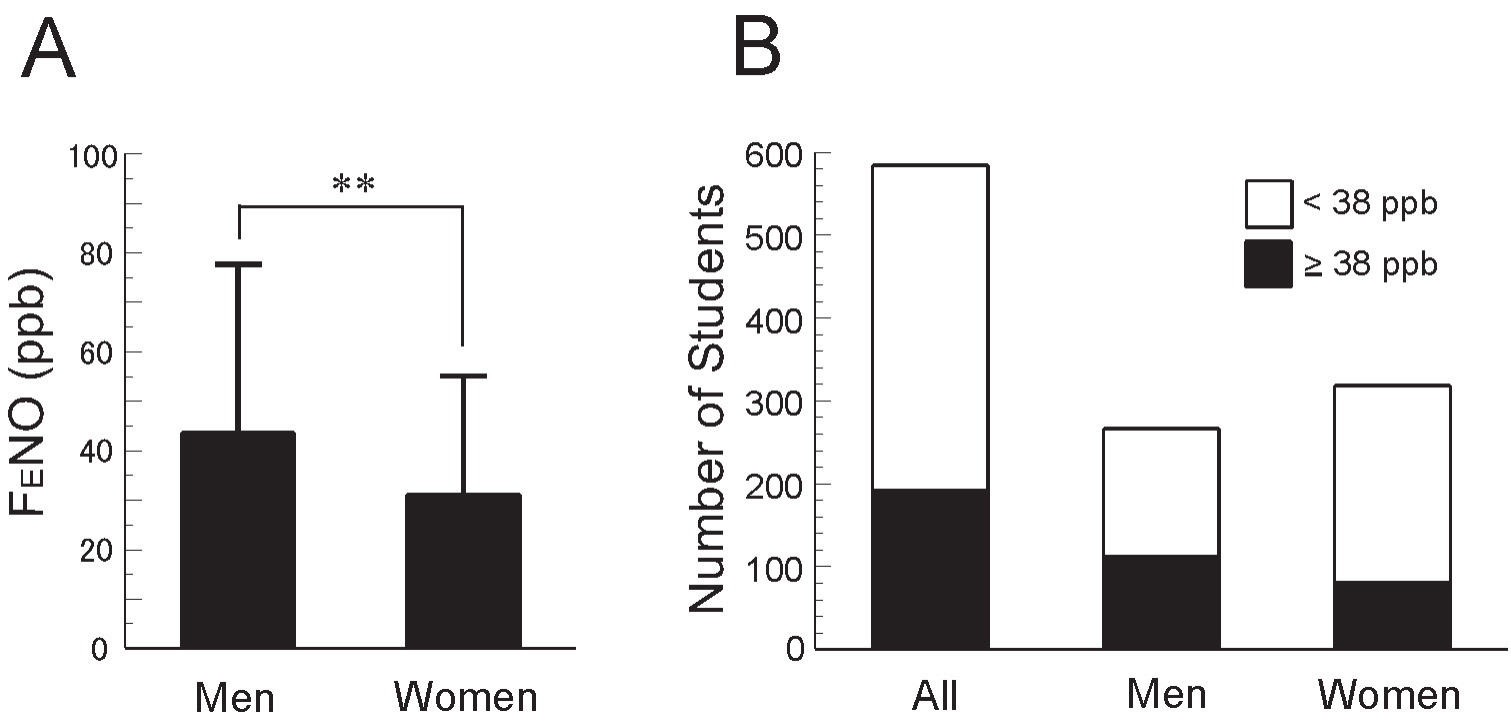
**A gender difference in FENO and the percentage of students whose FENO was ≥ 38.0 ppb**. In the 584 students ranging in age from 18 to 24 years, FENO was significantly higher in men than in women (**, *p *< 0.01) (A). Overall, 32.7% of the 584 students, including 41.2% of male students and 25.2% of female students, had FENO ≥ 38.0 ppb. The percentage of men was significantly higher than that of women (*p *< 0.01) (B).

The students ranged in age from 18 to 24. The mean ± SD FENO in 584 students was 36.7 ± 29.6 ppb, and was ≥ 38.0 ppb in 191 (32.7%) of 584 students. Among the 266 male students, the mean FENO was 43.6 ± 34.0 ppb, and was ≥ 38.0 ppb in 111 (41.7%) of 266 students. Among the 318 female students, the mean FENO was 31.0 ± 24.0 ppb, and was ≥ 38.0 ppb in 80 (25.2%) of 318 students. FENO levels and the percentage of students whose FENO was ≥ 38.0 ppb were significantly higher in men than in women (*p *< 0.01, Figure 2).

**Figure 3:**
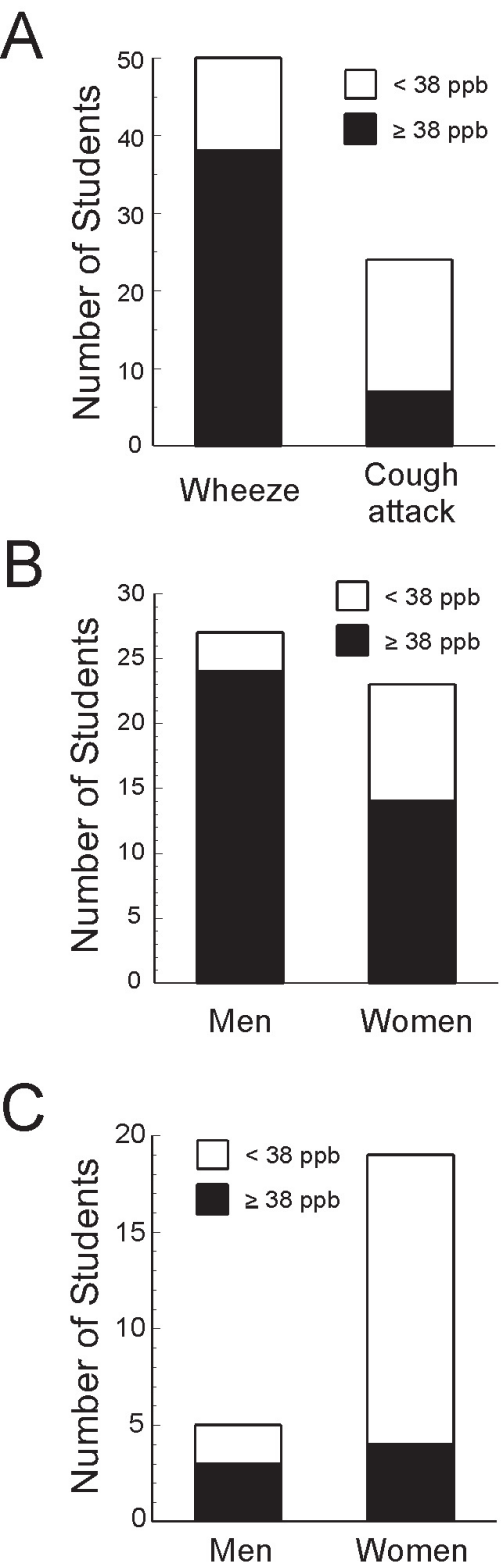
**The percentage of students with wheezes or coughs whose FENO was ≥ 38.0 ppb**. The percentage of students whose FENO was ≥ 38.0 ppb was higher in students with wheezes than in those with nocturnal attacks of cough (A). Among students with wheezes, FENO was ≥ 38.0 ppb in a significantly higher percentage of male than female students (*p *< 0.05) (B). Nocturnal attacks of cough were observed significantly more often in women than in men (*p *< 0.05). Among students with nocturnal attacks of cough, FENO was ≥ 38.0 ppb in a smaller percentage of female than male students (C).

FENO was ≥ 38.0 ppb in 38 (24 men and 14 women) of the 50 students (27 men and 23 women) who had experienced wheezing. It was ≥ 38.0 ppb in 7 (3 men and 4 women) of 24 students (5 men and 19 women) who had a nocturnal attack of cough. The percentages of subjects whose FENO was ≥ 38.0 ppb were 76.0% of the subjects who had wheezing and 29.2% of those with a nocturnal attack of cough. The percentage of students whose FENO was ≥ 38.0 ppb was smaller in those who had nocturnal attacks of cough than in those who had experienced wheezing (Figure 3A). The percentage of students whose FENO was ≥ 38.0 ppb in those who had experienced wheezing was 88.9% in men and 60.9% in women; the difference was significant (*p *< 0.05) (Figure 3B). Although a nocturnal attack of cough was relatively frequent in women, only 4 (21.1%) of 19 female students who had nocturnal attacks of cough had a FENO ≥ 38.0 ppb (Figure 3C).

### 2. Asthmatic symptoms in University students based on the ECRHS questionnaire

**Figure 4:**
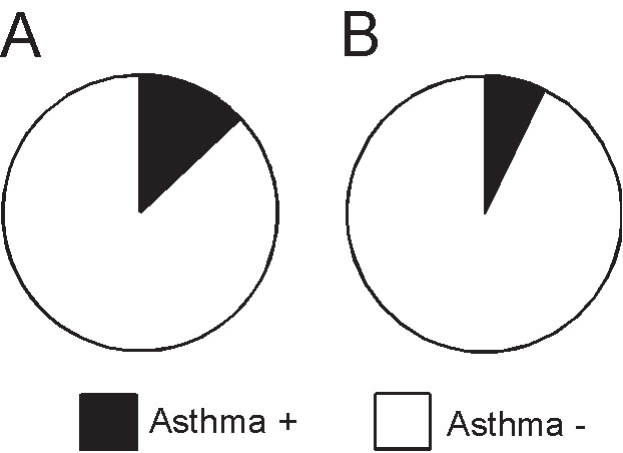
**The prevalence of asthma symptoms in students ranging in age from 18 to 24 years**. Overall, 77 (13.2%) of 584 students experienced any asthmatic symptoms within the last year. One-year prevalence of asthma symptoms in university students ranging in age from 18 to 24 years was expected to be 13.2% at a maximum (A). FENO was over 38.0 ppb in 42 of the 77 students. The prevalence of asthma symptoms was estimated to be 7.2% by considering FENO levels (B).

Of the 584 students, 77 (13.2%) answered that they had any asthmatic symptoms, including wheezing, a nocturnal feeling of tightness in the chest, a nocturnal attack of shortness of breath, or a nocturnal attack of cough in the last year (Figure 4A). Thus, the maximum prevalence of asthma symptoms in Gunma University students was 13.2%. Thirty-three (12.4%) of 266 male students and 44 (13.8%) of 318 female students had asthmatic symptoms. The percentage of students who had asthmatic symptoms was not significantly different between men and women. Of the 584 students, 50 (8.6%) reported wheezing, 11 (1.9%) had a nocturnal feeling of tightness in the chest, 6 (1.0%) had a nocturnal attack of shortness of breath, and 24 (4.1%) had a nocturnal attack of cough within the last year. Overall, 10.2% (27 of 266) of men and 7.2% (23 of 318) of women had wheezing. On the other hand, significantly more women (6.0%, 19 of 318) had a nocturnal attack of cough than men (1.9%, 5 of 266; *p *< 0.05).

### 3. The prevalence of asthma symptoms based on the ECRHS questionnaire and FENO measurement

FENO was over 38.0 ppb in 42 (25 men and 17 women) of the 77 students who had any asthmatic symptoms. Thus, the prevalence of asthma symptoms over 1 year in Gunma University students was 7.2% of 584 students, based on our definition of a predictive FENO and the information about symptoms obtained from the ECRHS questionnaire (Figure 4B). As a result of this analysis, the prevalence of asthma symptoms was greater in men (9.4%) than in women (5.3%).

### 4. Past history of asthma and current subjective symptoms

**Figure 5:**
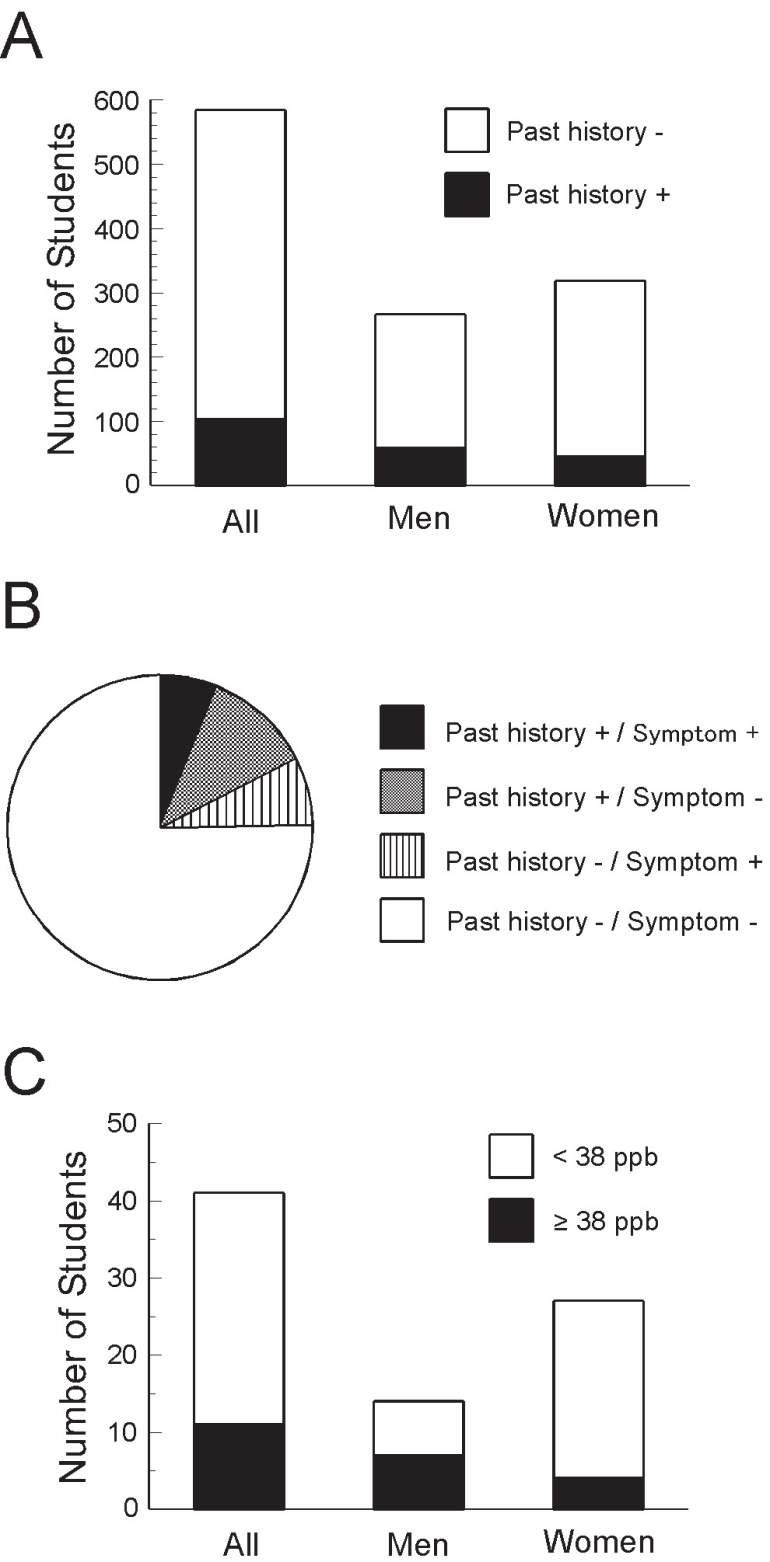
**The relationship between past history of asthma and current symptoms of asthma**. Overall, 17.6% of students ranging in age from 18 to 24 years had suffered from asthma. The percentage of students who had a past history of asthma was significantly greater in men (21.8%) than in women (14.2%) (*p *< 0.05) (A). Of the students who had suffered from asthma, 35% had current asthma symptoms. Further, 41 (7.0%) of 584 students ranging in age from 18 to 24 years had any asthmatic symptoms, including wheezing, a nocturnal feeling of tightness in the chest, a nocturnal attack of shortness of breath, or a nocturnal attack of cough, within the last year, although they did not have a past history of asthma (B). FENO was ≥ 38.0 ppb in 11 of 41 students who had any asthmatic symptoms within the last year, but did not have a past history of asthma. FENO was ≥ 38.0 ppb in 7 of 14 male students and 4 of 27 female students. The percentage of these 41 students whose FENO was ≥ 38.0 ppb was significantly greater in men than in women (*p *< 0.05) (C).

A total of 103 students (58 men and 45 women; 17.6% of 584 students) answered that they had suffered from asthma, including 21.8% of 266 male students and 14.2% of 318 female students; the difference between men and women was significant (*p *< 0.05) (Figure 5A). Thirty six (35.0%) of 103 students who suffered from asthma still had asthmatic symptoms (Figure 5B). Overall, 19 (32.8%) of 58 male students and 17 (37.8%) of 45 female students with a past history of asthma still felt any asthmatic symptoms. Of the 103 students who had suffered from asthma, 16 were being treated at the time of the survey, of whom only 3 had been treated well and had no asthmatic symptoms within the last year. Only 36.1% of the 36 students with current asthmatic symptoms and a past history of asthma were being treated medically at the time of the survey, and the other 63.9% were not being treated. A total of 41 students (14 men and 27 women) who did not have a past history of asthma and did not recognize themselves as having asthma had asthmatic symptoms within the last year. They accounted for 7.0% of the 584 students. Even if the students did not have a past history of asthma, they were also considered to have asthma when they had any asthmatic symptoms within the last year and their FENO was ≥ 38.0 ppb. FENO was ≥ 38.0 ppb in 11 (26.8%; 7 men and 4 women) of 41 students. A significantly greater percentage of men (50.0%) than women (14.8%) had a FENO ≥ 38.0 ppb (*p *< 0.05) (Figure 5C).

### 5. Nasal allergies including hay fever

Of the 584 students, 314 (53.8%) answered "Yes" to the question "Do you have any nasal allergies including hay fever?" FENO was significantly higher in students who had any nasal allergies including hay fever than in other students (41.4 ± 32.2 ppb vs. 31.3 ± 25.4 ppb, mean ± SD, *p *< 0.01). The percentage of students who had any nasal allergies did not differ between men (54.9%, 146 of 266) and women (52.8%, 168 of 318).

## Discussion

The gold standard of asthma diagnosis is obstructive lung disease with airway reversibility, or airway reactivity confirmed by challenge testing, not history or symptoms. Information obtained from the questionnaire which only asks asthmatic symptoms and history of asthma is not enough to make diagnosis of asthma, because clinical suspicion of asthma should be confirmed by objective measures of pulmonary function [20]. Strictly, current asthma symptoms on the ECRHS questionnaire are not equal to clinically current symptoms because this questionnaire is designed to detect asthma symptoms over 1 year. Although we must also emphasize that the ROC curve was constructed only to classify subjects of this study and that it was not constructed to help diagnose asthma, a previous study using the same offline method proposed that the optimal cut-off value of FENO was 38.0 ppb for detecting allergic airway inflammation in an adult population [10]. This cut-off value was equal to that we used in this study.

In our survey, two symptoms, wheeze and a nocturnal attack of cough were most frequently reported. FENO was ≥ 38.0 ppb in more than 75% of subjects who had wheeze. In those students who had experienced wheezing, the percentage whose FENO was ≥ 38.0 ppb was significantly higher in men than in women. This suggests that wheeze as a subjective symptom correlates with the presence of chronic eosinophilic airway inflammation especially in men. In fact, wheeze is the most sensitive and specific symptom for the diagnosis of asthma [21]. Although a few studies have described gender differences in asthmatic symptoms [22-24], a nocturnal cough attack was observed significantly more often in women than in men in our survey as well as a previous study in Australia [22]. Less than 30% of subjects who had a nocturnal attack of cough had an FENO ≥ 38.0 ppb. It was even smaller when subjects were limited to women. This suggests that a nocturnal cough attack as a subjective symptom is not closely related to the presence of chronic eosinophilic airway inflammation that is characteristic for typical asthma. Although a cough attack disturbing sleep is also one of the important symptoms that indicates asthma [22,25], it might be risky to make diagnosis of asthma based on this symptom especially in women.

In our study, FENO was significantly higher in men than in women aged between 18 to 24 years. Similarly, it has been reported that FENO was significantly higher in males than in females, in healthy children and healthy adults [26-29]. However, there are also some reports that deny the gender difference of FENO [30-35]. Thus, the influence of gender on FENO is still a matter of some controversy. Because gender difference in FENO was observed in our study, we tried to construct the ROC curve separately for males and females. When 44.0 ppb was used as the cut-off value for men and 37.0 ppb was for women, both sensitivity and specificity were much the same between men and women. We reanalyzed all data by using the gender-specific cut-off value to make assurance doubly sure. As a result, only one man was excluded from asthma and only one woman was included in asthma. Namely, the man who had experienced a cough attack was not considered to have asthma because his FENO was 38.4 ppb and the woman who had experienced wheezing was considered to have asthma because her FENO was 37.5 ppb. When we used the gender-specific cut-off value, FENO levels were the cut-off value and over in 34.8% of 266 male students and 26.1% of 318 female students. The percentage was significantly greater in men than in women (*p < 0.05*). Therefore, the results in our study did not change so much even if we used the gender-specific cut-off value.

In general, boys have a higher current prevalence of asthma than girls. This trend is reversed in adulthood, such that males have a lower prevalence than females. Actually, the percentage of students with a past history of asthma was higher in male students than in female students in our study. Gender differences in both FENO levels and a past history of asthma might mean that eosinophilic airway inflammation induced by prolongation of childhood asthma is currently present more in men than in women aged 18 to 24 years. Of students in this age group who had suffered from and still had asthma, only 3 students were treated well, and 36 students still had asthma symptoms within the last year, which suggests that this is a problem for patients in this age group. Furthermore, about two thirds of 36 students did not see a doctor at the time of our survey. We assume that at least 11 students who did not recognize themselves as having asthma probably did have asthma because their FENO levels were over 38.0 ppb and they had asthmatic symptoms within the last year. These students should be also recommended to see a doctor.

There are many other factors that can influence FENO levels [9]. In our study, many students had nasal allergy and it may affect FENO levels [36,37]. As expected, FENO was significantly higher in students who had any nasal allergies including hay fever than in other students. Interestingly, gender difference was not observed in the prevalence of nasal allergy on the basis of the questionnaire. Although Asian race may be a factor which increases FENO levels [38], most subjects were Japanese in this study.

The prevalence of asthma based on subjective asthmatic symptoms was 13.2% in university students ranging in age from 18 to 24 years when we estimated the maximum potential prevalence. The prevalence in female students exceeded that in male students by 1.4 percentage points. When the prevalence of asthma for the last year was estimated on the basis of FENO levels, as well as subjective asthmatic symptoms, it was 7.2%. Interestingly, the prevalence of asthma symptoms estimated by considering FENO in men exceeded that in women by 4.1 percentage points. This indicates that prevalence of asthma may be estimated more objectively by considering both asthma symptoms and the degree of eosinophilic airway inflammation. Patients with asthma in this age group need to be adequately diagnosed and treated well.

## Conclusions

The prevalence of asthma symptoms in university students in Japan in a single year was 7.2%, estimated on the basis of FENO levels as well as subjective asthmatic symptoms. The prevalence, based on both of these factors, was higher in men than in women. Gender differences were observed in both FENO levels and asthma symptoms.

## List of abbreviations

ECRHS: European Community Respiratory Health Survey; FENO: The fractional concentration of nitric oxide in exhaled air.

## Competing interests

The authors declare that they have no competing interests.

## Authors' contributions

Intellectual planning of the project was mainly done by TI. TH, KO, KD, TT, KA, and MM participated in the design of the study. Actual experimental works were performed by TI, SM, HA, MY, and YK. TH, TT, and KA were involved in the development of methods for FENO measurements. All authors read and approved the final manuscript.

## Authors' information

All authors are Doctor of Medicine. TI, TH, KD, and KA are representatives of the Japanese Society of Allergology. TI, SM, TH, TT, KD, and KA are medical specialists approved by the Japanese Society of Allergology. TI is an associate professor of Gunma University Graduate School of Medicine and a clinical professor of Gunma University Hospital. SM, HA, MY and YK are research fellows in Gunma University Graduate School of Medicine. TH is an assistant professor of Gunma University Graduate School of Medicine. TT is on the faculty of Sagamihara National Hospital. KD is a professor of Gunma University Graduate School of Health Sciences. KO is a professor of Health and Medical Center, Gunma University. KA is the chief executive officer of the Japanese Society of Allergology and the director of Sagamihara National Hospital. MM is a professor of Gunma University Graduate School of Medicine.

## Supplementary Material

Additional file 1 **The ROC curve constructed using FENO data in university students**. The ROC curve was constructed using FENO data obtained from 504 students in this study (including the samples obtained from students who were ≥ 25 years old). The asthmatic subjects included 38 students who answered that they suffered from asthma and had wheezing in the last year. The control subjects included 466 students who answered that they did not suffer from asthma and did not have any asthmatic symptoms, including wheezing, a nocturnal feeling of tightness in the chest, a nocturnal attack of shortness of breath, or a nocturnal attack of cough in the last year, and had not experienced chronic bronchitis-like symptoms, coughing and phlegm on most days for a minimum of 3 months a year and for at least 2 successive years. Click here for file
