# Supplementary figures and images for: Prevalence of asthma symptoms based on the European Community Respiratory Health Survey questionnaire and FENO in university students: gender differences in symptoms and FENO
Source: Allergy Asthma Clin Immunol. 2011 Sep 19;7(1):15. doi: 10.1186/1710-1492-7-15 (PMC3193803; doi:10.1186/1710-1492-7-15)

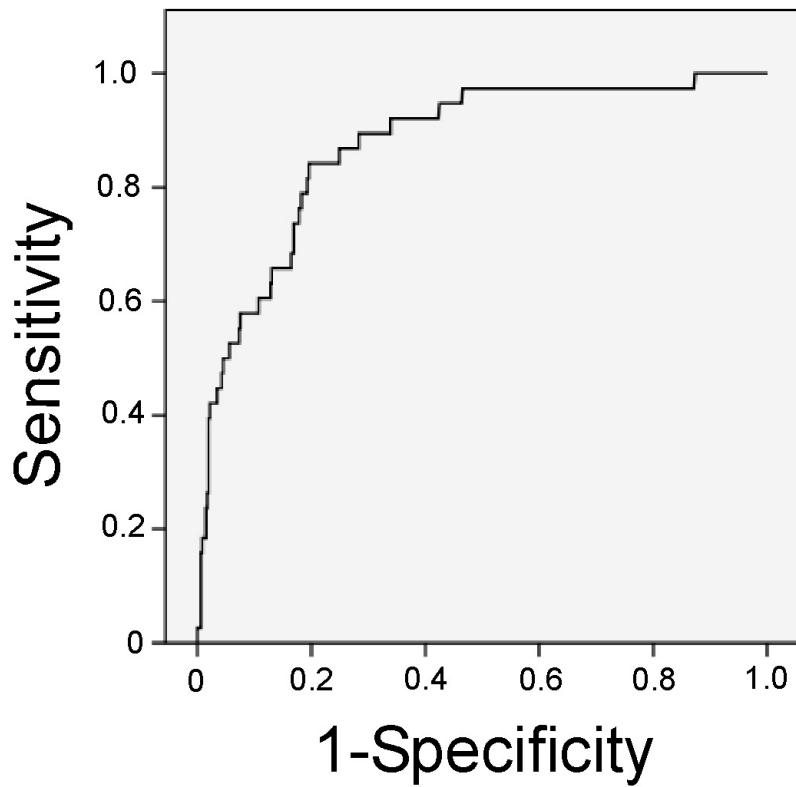

Supplement: Additional file 1 — The ROC curve constructed using FENO data in university students. The ROC curve was constructed using FENO data obtained from 504 students in this study (including the samples obtained from students who were ≥ 25 years old). The asthmatic subjects included 38 students who answered that they suffered from asthma and had wheezing in the last year. The control subjects included 466 students who answered that they did not suffer from asthma and did not have any asthmatic symptoms, including wheezing, a nocturnal feeling of tightness in the chest, a nocturnal attack of shortness of breath, or a nocturnal attack of cough in the last year, and had not experienced chronic bronchitis-like symptoms, coughing and phlegm on most days for a minimum of 3 months a year and for at least 2 successive years. [file 1710-1492-7-15-S1.PDF]
